# Risk of Relapse in Psychotic and Bipolar Disorders After Prenatal Antipsychotic Discontinuation

**DOI:** 10.1001/jamanetworkopen.2026.0682

**Published:** 2026-03-27

**Authors:** Xiaoqin Liu, Shelby Smout, Behrang Mahjani, Trine Munk-Olsen, Emely Ek Blæhr, Thalia K. Robakis, Veerle Bergink

**Affiliations:** 1 The National Centre for Register-based Research, Department of Public Health, Aarhus University, Aarhus, Denmark; 2 Department of Psychiatry, Icahn School of Medicine at Mount Sinai, New York, New York; 3 Research Unit of Child and Adolescent Psychiatry, Department of Clinical Research, University of Southern Denmark, Odense, Denmark; 4 Department of Psychiatry, Erasmus Medical Centre Rotterdam, Rotterdam, the Netherlands

## Abstract

**Importance:**

Antipsychotics are increasingly used in the perinatal period. However, evidence of their effectiveness in the perinatal population is limited.

**Objectives:**

To examine the risk of severe psychiatric relapse during the perinatal period associated with discontinuation of antipsychotic treatment before or during pregnancy in women with primary psychotic disorders or bipolar disorder.

**Design, Setting, and Participants:**

This cohort study used linked Danish and Swedish national birth registers to identify pregnancies leading to singleton live births from 1998 to 2022 in Denmark and from 2007 to 2017 in Sweden. Women diagnosed with psychotic disorders or bipolar disorder, with a gestational age between 154 and 315 days, and 2 or more antipsychotic prescriptions filled within 1 year before pregnancy were included. Data were analyzed from August 2024 to October 2025.

**Exposure:**

The exposure of interest was antipsychotic discontinuation. Discontinuation was defined as no prescription refill within the expected prescription duration plus a 60-day grace period. Women were classified into groups: prepregnancy discontinuation, pregnancy discontinuation, and continuation. The prepregnancy discontinuation group was matched 1:1 to the continuation group, and the pregnancy discontinuation group was separately matched 1:1 to the same continuation group.

**Main Outcomes and Measures:**

The outcome of interest was severe psychiatric relapse in the perinatal period. Hazard ratios (HRs) were estimated for psychiatric relapse, defined as inpatient treatment for psychiatric disorders during pregnancy and up to 3 months post partum, using a stratified Cox proportional hazards regression model.

**Results:**

A total of 2000 women with primary psychotic disorders (1265 from Denmark and 735 from Sweden) and 1292 women with bipolar disorder (341 from Denmark and 951 from Sweden) were included. The mean (SD) age was 30.8 (6.0) years for women with psychotic disorders and 29.1 (7.7) years for women with bipolar disorder. For women with psychotic disorders discontinuing antipsychotics before pregnancy, the adjusted HR (AHR) of psychiatric relapse was 1.24 (95% CI, 0.82-1.90) compared with continuation. Pregnancy discontinuation had a higher point estimate for relapse risk (328 pairs; AHR, 1.60 [95% CI, 1.01-2.54]) compared with continuation of antipsychotics. Among women with bipolar disorder, prepregnancy discontinuation (102 pairs; AHR, 0.50 [95% CI, 0.22-1.11]) and pregnancy discontinuation (92 pairs; AHR, 1.00 [95% CI, 0.48-2.10]) point estimates did not show an increased risk of severe relapse, but statistical power was limited.

**Conclusions and Relevance:**

In this cohort study, women with psychotic disorders who discontinued antipsychotic treatment during pregnancy were at a higher risk of severe relapse and the results for those who discontinued prepregnancy suggest a possible increase in risk, compared with those who continued, which aligns with findings observed beyond the perinatal period; results in women with bipolar disorder were inconclusive. There is an urgent need to investigate the effectiveness of antipsychotics on both severe and nonsevere relapses in the perinatal period in prospective clinical cohort studies.

## Key Points

**Question:**

Is discontinuing antipsychotics before or during pregnancy associated with increased risk of severe psychiatric relapse?

**Findings:**

In this cohort study of 2000 women with primary psychotic disorders and 1292 women with bipolar disorder in Denmark and Sweden, discontinuation of antipsychotics during pregnancy was associated with a higher relapse risk among women with psychotic disorders. For women with bipolar disorder, sufficient statistical power was lacking to conclude whether discontinuation was associated with relapse risk.

**Meaning:**

The findings of this study suggest a need to investigate the effectiveness of antipsychotics on both severe and nonsevere relapses in the perinatal period.

## Teaser

This cohort study explores the association between antipsychotic medication discontinuation before or during pregnancy and severe psychiatric relapse in the perinatal period.

## Introduction

Antipsychotics are increasingly prescribed to women of childbearing age.^1^ Estimates show that 0.3% to 4.6% of pregnancies involve women taking antipsychotics.^2^ Antipsychotics are prescribed mainly for psychotic and bipolar disorders but have also been used as augmentation for unipolar depression and obsessive-compulsive disorder as well as off-label medication for anxiety and sleep problems.^3^ The effectiveness of antipsychotics for the management of psychotic disorders, such as schizophrenia; schizoaffective disorder; and conditions with hallucinations, delusions, or severe thought disturbances, has been confirmed through extensive research and meta-analyses.^4,5^ For bipolar disorder, antipsychotics’ effectiveness has been shown for the treatment of mania and depressive episodes, while evidence of beneficial outcome for maintenance treatment has been found for specific agents in some studies but not in others.^6,7,8,9^

Despite growing use, more than half of the women in studies discontinued antipsychotic treatment around conception,^10^ likely due to concerns about fetal risks. While antipsychotics are well-established treatments for psychotic and bipolar disorders in the general population,^4,5,6,7,8,9^ evidence supporting antipsychotics’ effectiveness specifically during the perinatal period remains limited. A South Korean population-based study found that women with schizophrenia who continued antipsychotics had a 56% lower risk of postpartum relapse compared with participants who discontinued.^11^ However, a UK study of women with severe mental illness found no protective benefit from continued medication.^12^ These conflicting findings underscore the uncertainty clinicians face when counseling pregnant women about antipsychotic continuation.

The perinatal period presents unique considerations for psychiatric treatment, given that pregnancy and childbirth are associated with substantial changes in physiological functions, circadian rhythm, and interpersonal circumstances. Additionally, drug metabolism and distribution change during the perinatal period.^13,14^ While pregnancy appears protective against first-onset psychosis and mania,^15^ the postpartum period carries substantially elevated risks for severe psychiatric episodes.^16^ These complex factors suggest that treatment outcomes observed in general populations may not directly translate to perinatal contexts, necessitating specific investigation of antipsychotics’ effectiveness during pregnancy. In this study, we aimed to examine the risk of severe psychiatric relapse during the perinatal period (ie, throughout pregnancy until 3 months after delivery) associated with discontinuation of antipsychotic treatment before or during pregnancy in women with primary psychotic disorders or bipolar disorder.

## Methods

### Study Population

We conducted a population-based propensity score–matched cohort study linking national registers in Denmark and Sweden. The Danish Data Protection Agency and the Regional Ethical Review Board in Stockholm, Sweden, approved this study. In accordance with laws in Denmark and Sweden, informed consent was not required for register-based studies using anonymized data. We followed the Strengthening the Reporting of Observational Studies in Epidemiology (STROBE) reporting guideline.

All live-born children and new residents in both countries are assigned a unique personal identification number, which can be used to link the data between and within registers. We identified pregnancies leading to singleton live births between January 1, 1998, and September 30, 2022, from the Danish Medical Birth Register and between January 1, 2007, and December 31, 2017, from the Swedish Medical Birth Register. We focused on pregnant women meeting the following criteria: (1) gestational age between 154 and 315 days, (2) fill of at least 2 antipsychotic prescriptions within 365 days prior to the start of pregnancy, and (3) diagnosis of bipolar disorder or primary psychotic disorders before the first index antipsychotic prescription. We estimated the start of pregnancy based on the ultrasonography scan; if these scans were unavailable, the first day of the last menstrual period was used.^17,18^

### Exposure 

The exposure of interest was antipsychotic discontinuation. Data on antipsychotic use were retrieved from the Danish National Prescription Registry and the Swedish Prescribed Drug Register, using the anatomical therapeutic chemical (ATC) classification code N05A, excluding lithium (ATC code N05AN01). Antipsychotic continuation was defined as consistent filling of prescriptions from the second prescription redeemed within 365 days before pregnancy through delivery. Continuous treatment was defined as the supply duration of each prescription plus a 60-day grace period. Switching to a different antipsychotic medication during this time frame was still considered continuous treatment. Antipsychotic discontinuation was defined as the absence of a new prescription within this time frame. Based on these criteria, included women were categorized as follows: (1) continuation group, which maintained antipsychotics throughout the prenatal period; (2) prepregnancy discontinuation group, which stopped antipsychotics before pregnancy; and (3) pregnancy discontinuation group, which stopped antipsychotics during pregnancy.

### Outcome 

The outcome of interest was severe psychiatric relapse in the perinatal period. We defined severe psychiatric relapse as inpatient treatment with a psychiatric disorder as the primary diagnosis (*International Statistical Classification of Diseases and Related Health Problems, Tenth Revision* [*ICD-10*] codes F00-F99) during pregnancy and within 90 days after delivery, excluding organic mental disorders (*ICD-10* codes F00-F09) and intellectual disability (*ICD-10* codes F70-F79) (eTable 1 in Supplement 1). We specifically examined the risk of postpartum relapse within 90 days after delivery, as findings from a previous study indicated that this risk is most important during this timeframe and decreases thereafter.^15^

Using directed acyclic graphs, we considered a priori a broad range of covariates that were potentially prognostically important for severe psychiatric relapse.^19^ These covariates included maternal demographics (age, marital status, and educational level), health status (primiparity, smoking during pregnancy, and prepregnancy body mass index [calculated as weight in kilograms divided by height in meters squared]), history of other psychiatric disorders (substance use disorder; depression; other mood disorders; neurotic, stress-related, and somatoform disorders; personality disorders; and other mental illnesses), coprescribed medication (lithium, antidepressants, anxiolytics and benzodiazepines, antiseizure medications, and opioids), and health care utilization within 12 months before pregnancy (inpatient or emergency department [ED] visit and outpatient visit for psychiatric disorders). We further included the calendar year of delivery to account for the calendar factor. eFigure 1 in Supplement 1 provides the graphical depiction of the timeline for assessing exposure, outcomes, and covariates.

### Statistical Analysis

We analyzed the data from Denmark and Sweden separately, using a harmonized protocol. Relative risk estimates from the 2 countries were pooled using random-effects meta-analytic models.^20^

All analyses were conducted separately for women with primary psychotic disorders and those with bipolar disorder. We calculated propensity scores using logistic regression to model the estimated probability of antipsychotic discontinuation vs continuation, based on all aforementioned variables. Specifically, we first estimated propensity score values for women in the prepregnancy discontinuation group vs the full continuation group. We then estimated a second set of propensity scores for women in the pregnancy discontinuation group vs the same continuation group (eFigures 2 and 3 in Supplement 1 show the distribution of propensity scores). We excluded any observations with propensity scores that did not overlap between discontinuation and continuation groups. Using these propensity scores, the continuation group was matched 1:1 to the discontinuation groups using nearest neighbor matching without replacement within a caliper width of 0.1. Matching was performed separately for the 2 discontinuation groups, using the respective propensity scores for each comparison. We chose a tighter caliper width over the recommended width of 0.2^21^ because a tighter caliper leads to substantially reduced bias,^22^ and the increase in the number of matched pairs using a width of 0.2 in this study was negligible.

For the prepregnancy discontinuation group and their matched continuation group, follow-up began on the first day of pregnancy. For the pregnancy discontinuation group, follow-up began on the date of discontinuation. Women in the continuation group matched to the pregnancy discontinuation group were followed up from the equivalent time point relative to the start of pregnancy, similar to their matched discontinuation group (eFigure 4 in Supplement 1). Follow-up ended at severe psychiatric relapse, death, emigration, 90 days after childbirth, or the end of the study, whichever occurred first.

We calculated standardized mean differences (SMDs) to assess covariate balance before and after propensity score matching (PSM) between groups. Meaningful imbalances were defined as an absolute standardized difference of more than 0.1.^23^ Stratified Cox proportional hazards regression model^24^ was used to estimate the hazard ratios (HRs) and 95% CIs. Each matched pair constituted a separate stratum, and each stratum had its own baseline hazard function.

To test the robustness of our results, we conducted 2 sensitivity analyses. First, we repeated the analyses in Denmark, defining severe psychiatric relapse as an inpatient or ED visit (eMethods in Supplement 1). Second, we investigated the relapse risk estimates, using a 30-day grace period to define antipsychotic discontinuation and continuation. When interpreting the results, we focused on the magnitude of the estimates in the context of their potential clinical and public health relevance, regardless of whether the 95% CI included the null. Data management and statistical analyses were performed from August 2024 to October 2025 using Stata 16.0 (StataCorp LLC) and R 4.0.5 (R Project for Statistical Computing).

## Results

**Figure 1.**
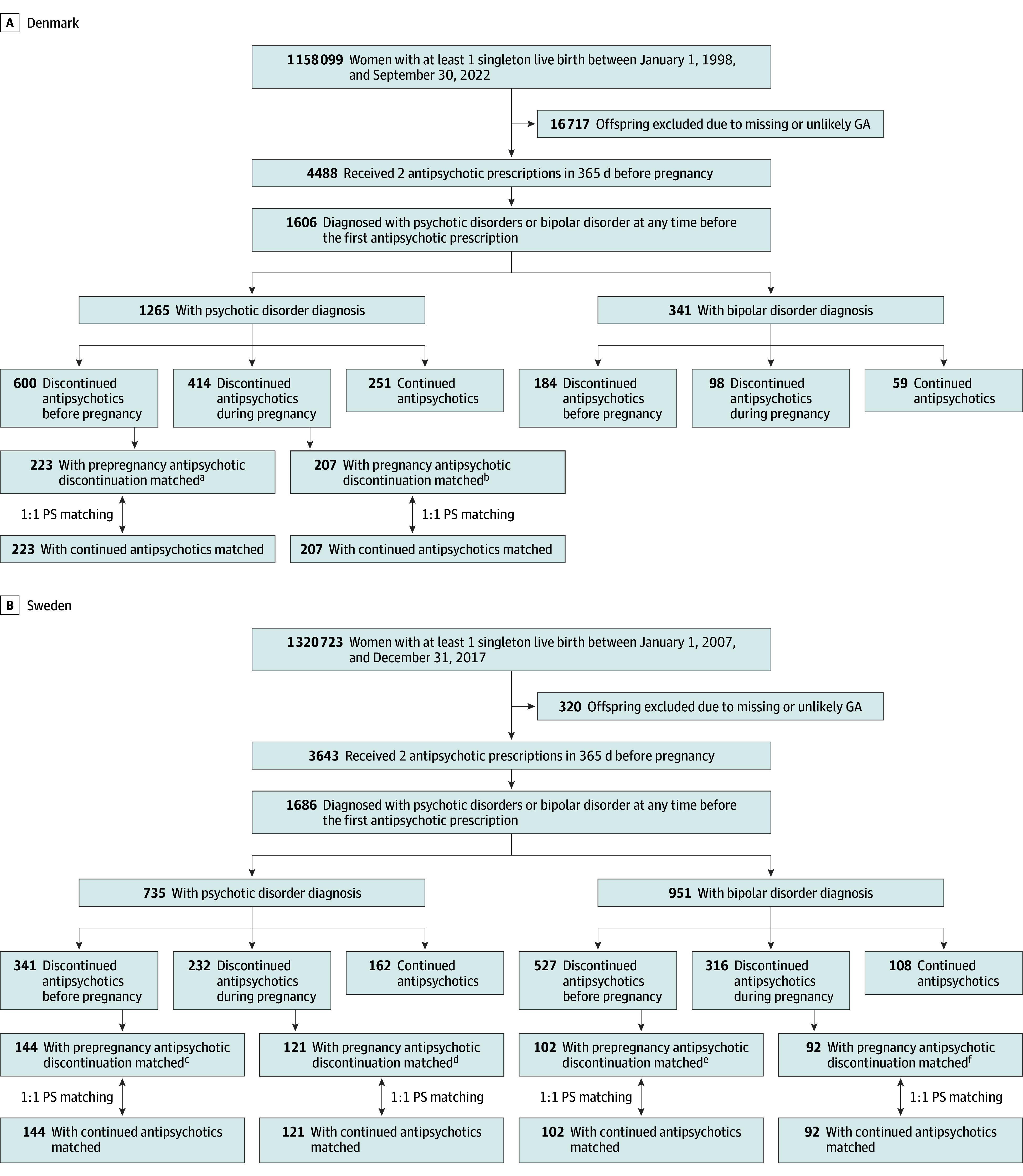
Flowchart of the Study Population Identification GA indicates gestational age; PS, propensity score. ^a^377 Women with psychotic disorders in Denmark could not be matched to women who continued antipsychotics. There were not enough women with bipolar disorder for matched analysis. ^b^207 Women with psychotic disorders in Denmark could not be matched to women who continued antipsychotics. There were not enough women with bipolar disorder for matched analysis. ^c^197 Women with psychotic disorders in Sweden could not be matched to women who continued antipsychotics. ^d^111 Women with psychotic disorders in Sweden could not be matched to women who continued antipsychotics. ^e^425 Women with bipolar disorder in Sweden could not be matched to women who continued antipsychotics. ^f^224 Women with bipolar disorder in Sweden could not be matched to women who continued antipsychotics.

Of the pregnancies with singleton live births identified from the Danish Medical Birth Register (n = 1 158 099) and the Swedish Medical Birth Register (n = 1 320 723), 1606 and 1686, respectively, were eligible for the analyses (Figure 1). The study included 2000 women with primary psychotic disorders (1265 from Denmark and 735 from Sweden) and 1292 women with bipolar disorder (341 from Denmark and 951 from Sweden). The mean (SD) age was 30.8 (6.0) years for women with psychotic disorders and 29.1 (7.7) years for women with bipolar disorder. Women with psychotic disorders were less likely to discontinue antipsychotics before or during pregnancy than women with bipolar disorder. Among women with psychotic disorders, 80.2% (1014 of 1265) in Denmark and 78.0% (573 of 735) in Sweden discontinued antipsychotic treatment before or during pregnancy. The corresponding proportions among women with bipolar disorder were 82.7% (282 of 341) in Denmark and 88.6% (843 of 951) in Sweden (Figure 1). The characteristics of the study populations are presented in Table 1 and Table 2.

**Table 1.** Characteristics of the Study Population by Antipsychotic Discontinuation Before Matching Among Women With Psychotic Disorders

Characteristic	Women in Denmark, No. (%)			Women in Sweden, No. (%)		
	Prepregnancy antipsychotic discontinuation (n = 600)	Pregnancy antipsychotic discontinuation (n = 414)	Antipsychotic continuation (n = 251)	Prepregnancy antipsychotic discontinuation (n = 341)	Pregnancy antipsychotic discontinuation (n = 232)	Antipsychotic continuation (n = 162)
Age, mean (SD), y	29.4 (5.7)	29.4 (6.0)	31.7 (5.5)	31.9 (5.8)	31.9 (6.0)	33.8 (5.8)
Primiparity	370 (61.7)	245 (59.2)	145 (57.8)	179 (52.5)	124 (53.5)	76 (46.9)
History of other mental disorders before pregnancy						
SUD	116 (19.3)	82 (19.8)	45 (17.9)	75 (22.0)	61 (26.3)	38 (23.5)
Depression	264 (44.0)	173 (41.8)	98 (39.0)	148 (43.0)	119 (51.3)	67 (41.4)
Other mood disorders	27 (4.5)	15 (3.6)	5 (2.0)	25 (7.3)	13 (5.6)	17 (10.5)
Neurotic, stress-related, and somatoform disorders	335 (55.8)	224 (54.1)	123 (49.0)	177 (51.9)	130 (56.0)	79 (48.8)
Personality disorders	229 (38.2)	176 (42.5)	92 (36.7)	75 (22.0)	49 (21.1)	31 (19.1)
Child-onset disorders	86 (14.3)	62 (15.0)	18 (7.2)	16 (4.7)	10 (4.3)	7 (4.3)
Other mental illnesses	130 (21.7)	117 (28.3)	65 (25.9)	51 (15.0)	35 (15.1)	12 (7.4)
Psychiatric inpatient or ED visit in 12 mo before pregnancy	140 (23.3)	140 (33.8)	57 (22.7)	29 (8.5)	37 (15.9)	11 (6.8)
Psychiatric outpatient visit in 12 mo before pregnancy	269 (44.8)	206 (49.8)	110 (43.8)	302 (88.6)	209 (90.1)	139 (85.8)
Coprescribed medications in 12 mo before pregnancy						
Opioids	61 (10.2)	53 (12.8)	24 (9.6)	29 (8.5)	25 (10.8)	13 (8.0)
Antidepressants	267 (44.5)	213 (51.4)	120 (47.8)	168 (49.3)	120 (51.7)	72 (44.4)
Lithium	16 (2.7)	15 (3.6)	5 (2.0)	17 (5.0)	10 (4.3)	15 (9.3)
Anxiolytics and benzodiazepines	171 (28.5)	120 (29.0)	59 (23.5)	174 (51.0)	141 (60.8)	86 (53.1)
Antiseizure medications	107 (17.8)	74 (17.9)	58 (23.1)	53 (15.5)	50 (21.6)	36 (22.2)
Smoking during pregnancy	231 (38.5)	184 (44.4)	112 (44.6)	24 (7.0)	19 (8.2)	19 (11.7)
Prepregnancy BMI ≥25	267 (44.5)	187 (45.2)	142 (56.6)	195 (57.2)	144 (62.1)	115 (71.0)
Maternal marital or cohabiting status in the year of pregnancy	429 (71.5)	271 (65.5)	184 (73.3)	244 (71.6)	175 (75.4)	105 (64.8)
Maternal highest educational level in the year of pregnancy						
Mandatory school	340 (56.7)	240 (58.0)	131 (52.2)	88 (25.8)	57 (24.6)	38 (23.5)
High school or vocational school	159 (26.5)	99 (23.9)	81 (32.3)	125 (36.7)	111 (47.8)	84 (51.9)
College or university	94 (15.7)	66 (15.9)	39 (15.5)	116 (34.0)	52 (22.4)	35 (21.6)
Missing data	7 (1.2)	9 (2.2)	0	12 (3.5)	12 (5.2)	5 (3.1)
Calendar year at the start of pregnancy						
1997-2004	99 (16.5)	78 (18.8)	40 (15.9)	0	0	0
2005-2010	146 (24.3)	118 (28.5)	63 (25.1)	139 (40.8)	94 (27.6)	68 (42.0)
2011-2015	157 (26.2)	97 (23.4)	44 (17.5)	152 (44.6)	99 (29.0)	71 (43.8)
2016-2022	198 (33.0)	121 (29.2)	104 (41.4)	50 (14.7)	39 (11.4)	23 (14.2)

Abbreviations: BMI, body mass index (calculated as weight in kilograms divided by height in meters squared); ED, emergency department; SUD, substance use disorder.

**Table 2.** Characteristics of the Study Population by Antipsychotic Discontinuation Before Matching Among Women With Bipolar Disorder

Characteristic	Women in Denmark, No. (%)			Women in Sweden, No. (%)		
	Prepregnancy antipsychotic discontinuation (n = 184)	Pregnancy antipsychotic discontinuation (n = 98)	Antipsychotic continuation (n = 59)	Prepregnancy antipsychotic discontinuation (n = 527)	Pregnancy antipsychotic discontinuation (n = 316)	Antipsychotic continuation (n = 108)
Age, mean (SD), y	24.8 (10.9)	25.3 (10.6)	26.8 (9.9)	30.4 (5.9)	30.2 (5.6)	31.1 (5.2)
Primiparity	104 (56.5)	58 (59.2)	36 (61.0)	300 (56.9)	168 (53.2)	52 (48.1)
History of other mental disorders before pregnancy						
SUD	31 (16.8)	19 (19.4)	6 (10.2)	166 (31.5)	122 (38.6)	38 (35.2)
Depression	102 (55.4)	51 (52.0)	29 (49.2)	298 (56.5)	196 (62.0)	65 (60.2)
Other mood disorders	6 (3.3)	13 (13.3)	5 (8.5)	64 (12.1)	34 (10.8)	5 (4.6)
Neurotic, stress-related, and somatoform disorders	85 (46.2)	52 (53.1)	31 (52.5)	360 (68.3)	231 (73.1)	66 (61.1)
Personality disorders	62 (33.7)	39 (39.8)	21 (35.6)	176 (33.4)	106 (33.5)	39 (36.1)
Child-onset disorders	22 (12.0)	13 (13.3)	8 (13.6)	38 (7.2)	22 (7.0)	4 (3.7)
Other mental illnesses	43 (23.4)	25 (25.5)	11 (18.6)	162 (30.7)	85 (26.9)	26 (24.1)
Psychiatric inpatient or ED visit in 12 mo before pregnancy	38 (20.7)	23 (23.5)	6 (10.2)	42 (8.0)	32 (10.1)	16 (14.8)
Psychiatric outpatient visit in 12 mo before pregnancy	83 (85.1)	55 (56.1)	33 (55.9)	479 (90.9)	294 (93.0)	101 (93.5)
Coprescribed medications in 12 mo before pregnancy						
Opioids	21 (11.4)	10 (10.2)	<5	104 (19.7)	53 (16.8)	18 (16.7)
Antidepressants	78 (42.4)	44 (44.9)	20 (33.9)	309 (58.6)	200 (63.3)	65 (60.2)
Lithium	50 (27.2)	14 (14.3)	10 (16.9)	156 (29.6)	91 (28.8)	42 (38.9)
Anxiolytics and benzodiazepines	36 (19.6)	27 (27.6)	16 (27.1)	355 (67.4)	225 (71.2)	79 (73.1)
Antiseizure medications	109 (59.2)	59 (60.2)	29 (49.2)	284 (53.9)	177 (56.0)	51 (47.2)
Smoking during pregnancy	58 (31.5)	37 (37.8)	16 (27.1)	60 (11.4)	47 (14.9)	13 (12.0)
Prepregnancy BMI ≥25	84 (45.7)	40 (40.8)	29 (49.2)	281 (53.3)	168 (53.2)	79 (73.1)
Maternal marital or cohabiting status in the year of pregnancy	125 (67.9)	75 (76.5)	44 (74.6)	405 (76.9)	227 (71.8)	86 (79.6)
Maternal highest educational level in the year of pregnancy						
Mandatory school	53 (28.8)	33 (33.7)	15 (25.4)	85 (16.1)	72 (22.8)	21 (19.4)
High school or vocational school	75 (40.8)	36 (36.7)	26 (44.1)	272 (51.6)	159 (50.3)	51 (47.2)
College or university	56 (30.4)	29 (29.6)	18 (30.5)	157 (29.8)	74 (23.4)	34 (31.5)
Missing data	0	0	0	13 (2.5)	11 (3.5)	2 (1.9)
Calendar year at the start of pregnancy^a^						
1997-2004	8 (4.4)	0	0	0	0	0
2005-2010	16 (8.7)	27 (27.6)	5 (8.4)	105 (19.9)	64 (20.3)	18 (16.7)
2011-2015	60 (32.6)	24 (24.5)	10 (16.9)	305 (57.9)	179 (56.6)	61 (56.5)
2016-2022	100 (54.4)	47 (48.0)	44 (74.6)	117 (22.2)	73 (23.1)	29 (26.9)

Abbreviations: BMI, body mass index (calculated as weight in kilograms divided by height in meters squared); ED, emergency department; SUD, substance use disorder.

^a^ Fewer than 5 women with bipolar disorder in Denmark gave birth between 1997 and 2004, and they were merged with the group who gave birth between 2005 and 2010.

After PSM, the characteristics were well balanced between groups among women with psychotic disorders, with SMDs below 0.1. For women with bipolar disorder, sufficient statistical power was available only in the Swedish cohort. Most characteristics were comparable between groups, except for a slightly lower prevalence of depression in the prepregnancy discontinuation group compared with their matched continuation group (SMD = 0.12). Additionally, prepregnancy body mass index was higher among the pregnancy discontinuation group than their matched continuation group (SMD = −0.11) (eFigures 5 and 6 in Supplement 1).

### Discontinuation and Relapse Among Women With Psychotic Disorders

**Figure 2.**
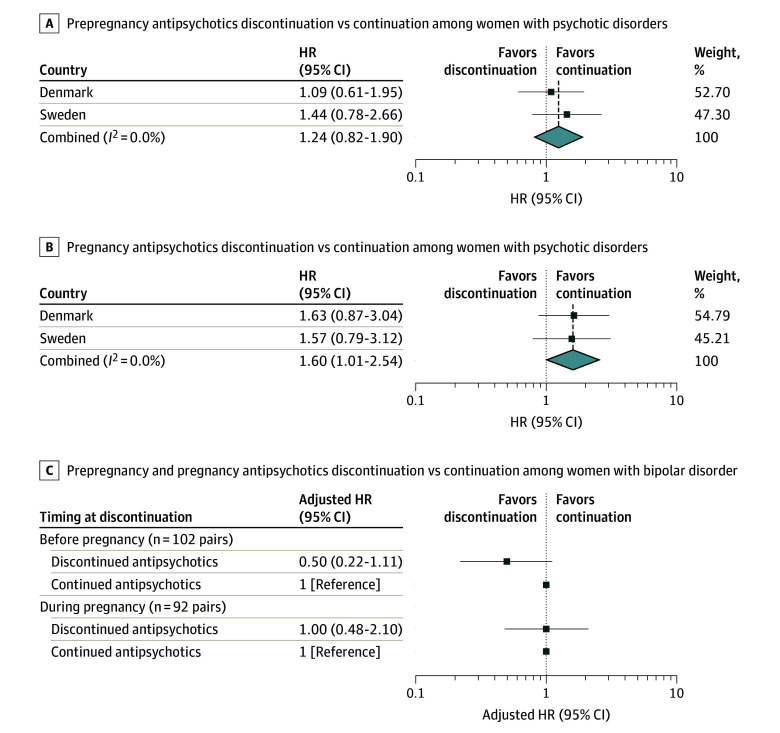
Forest Plots of Antipsychotic Discontinuation Before or During Pregnancy and Risk of Perinatal Severe Psychiatric Relapse HR indicates hazard ratio.

Among women with psychotic disorders, 367 who discontinued antipsychotics before pregnancy and 328 who discontinued during pregnancy were matched to women who continued treatment. Prepregnancy discontinuation had an adjusted HR (AHR) of 1.24 (95% CI, 0.82-1.90; *I*^2^  = 0.0%) for severe psychiatric relapse compared with continuation of antipsychotics. For pregnancy discontinuation, the AHR for relapse risk was 1.60 (95% CI, 1.01-2.54; *I*^2^ = 0.0%) compared with continuation of antipsychotics (Figure 2A and B; eTables 2 and 3 in Supplement 1).

### Discontinuation and Relapse Among Women With Bipolar Disorder

Among women with bipolar disorder, 102 who discontinued antipsychotics before pregnancy and 92 who discontinued during pregnancy were matched to women who continued treatment. The AHR for discontinuing antipsychotics before pregnancy was 0.50 (95% CI, 0.22-1.11) for the risk of severe psychiatric relapse, however, the 95% CIs were wide. In contrast, the AHR for relapse risk of discontinuing antipsychotics during pregnancy was 1.00 (95% CI, 0.48-2.10) (Figure 2C; eTables 2 and 3 in Supplement 1).

### Sensitivity Analysis

For women with psychotic disorders, redefining severe relapse in Denmark to include inpatient and ED visits shifted the HR for prepregnancy discontinuation from 1.09 (95% CI, 0.61-1.95) to 0.91 (95% CI, 0.55-1.50), with a smaller point estimate for pregnancy discontinuation (AHR, 1.30; 95% CI, 0.73-2.33) (eTable 4 in Supplement 1). Using an alternative 30-day grace period for defining discontinuation produced similar findings for women with bipolar disorder. For women with psychotic disorders, prepregnancy discontinuation of antipsychotics had an AHR of 1.36 (95% CI, 0.78-2.36) for relapse risk (eTable 5 in Supplement 1), whereas discontinuation during pregnancy had an AHR of 0.88 (95% CI, 0.49-1.58) (eTable 6 in Supplement 1). However, the estimate for discontinuation of antipsychotics during pregnancy was based on a small sample size, resulting in imprecise and unstable estimates.

## Discussion

Using nationwide registry data from Denmark and Sweden, we found that approximately 80% of women with primary psychotic disorders discontinued antipsychotic treatment before or during pregnancy, with even higher discontinuation rates among women with bipolar disorder. In women with psychotic disorders, discontinuation of antipsychotics during pregnancy was associated with an increased risk of relapse of about 60%, whereas prepregnancy discontinuation showed a lower tendency in the same direction. In contrast, among women with bipolar disorder, discontinuation did not appear to increase relapse risk; if anything, stopping treatment before pregnancy was associated with a lower risk, although estimates were imprecise due to limited statistical power.

Our findings for women with psychotic disorders align with evidence from both the general population^5,25^ and a recent Korean nationwide study examining postpartum relapse in women with schizophrenia.^11^ Kang et al^11^ reported a 44% relative risk reduction (relative risk ratio, 0.56; 95% CI, 0.36-0.87) among the antipsychotic continuation group compared with the discontinuation group, consistent with our findings. Important methodological differences exist between studies: we examined perinatal relapse using between-individual PSM, while Kang et al^11^ performed within-individual comparisons focused on the postpartum period and restricted to schizophrenia rather than the broader spectrum of psychotic disorders in the present study. The more pronounced risk with discontinuation of antispsychotics during pregnancy compared with prepregnancy discontinuation likely reflects differences in pregnancy planning and illness stability. Women in the prepregnancy discontinuation group may have stopped treatment as part of planned conception, potentially indicating greater baseline stability. This pattern of higher risk with discontinuation during vs before pregnancy has been documented for other psychotropic medications, including antidepressants,^26^ suggesting a broader phenomenon in perinatal psychiatry. Kang et al^11^ found no significant difference according to discontinuation timing, which contrasts with our findings. This difference could be due to their use of within-individual comparison, which would eliminate baseline differences in stability as a contributing factor.

For bipolar disorder, we did not find evidence of the benefits of antipsychotics against severe relapse, a finding that stands in contrast to numerous publications documenting the effectiveness of lithium in this population. Lithium is the mainstay of acute and maintenance treatment in bipolar disorder.^9,27^ Its effectiveness in reducing relapse risk in women with bipolar disorder in the perinatal period has been verified in a study^28^ and a meta-analysis.^29^ The retrospective cohort study showed a 40% relapse risk in participants without lithium vs 24% in participants with lithium (odds ratio, 0.47; 95% CI, 0.27-0.81).^28^ Moreover, the meta-analysis shows significantly higher postpartum relapse rates in medication-free women (66%, 95% CI, 57%-75%) than in those receiving prophylactic treatment, primarily lithium (23%; 95% CI, 14%-37%).^29^ These studies on the beneficial outcomes of lithium were all clinical cohort studies, which enable better detection of relapse. Results of these previous studies cannot be compared with findings of the present work, which used register-based data. Lithium is less widely used worldwide to treat bipolar disorder, partly due to a lack of confidence among clinicians. During the pregnancy period, there is a teratogenicity risk in the first trimester if lithium is used in higher doses, which might have led to a further decline in lithium use among women of reproductive age.^30^

Instead, antipsychotics have increasingly become the primary pharmacotherapy for pregnant women with bipolar disorder,^2,31^ despite minimal evidence supporting their effectiveness in the perinatal period. Existing literature is limited to 2 small case series (combined n = <25) examining only postpartum prophylaxis.^32,33^ Several factors urge us to interpret cautiously our null findings in women with bipolar disorder. First, statistical power was limited by the relatively small number of women who continued treatment. Historically, clinical practice has favored discontinuing antipsychotics before pregnancy due to safety concerns, substantially limiting the pool of women who continued treatment. This pattern is reflected in our cohort composition, where most women in the continuation group in both countries gave birth after 2011, when larger pharmacovigilance studies began providing reassuring safety data. Prior to this period, most women were likely counseled to discontinue antipsychotics when planning for pregnancy, explaining both our small sample of women who continued treatment and the temporal distribution of our data. Second, depressive relapses, which are common in bipolar disorder, do not often lead to inpatient admission or ED visits. This nondifferential misclassification would have biased the results toward the null. Third, residual confounding could theoretically bias the protective benefits toward the null if those who continued treatment had more severe illness, although this seems less plausible given that we observed substantial protection using similar methods in psychotic disorders.

### Clinical Implications

For women with psychotic disorders, our findings together with those of Kang et al^11^ provide converging evidence that continuing antipsychotic treatment during pregnancy is associated with reduced risk of severe psychiatric relapse. There is no such evidence for women with bipolar disorder during pregnancy. With regards to preconception consultation, if women decide to taper antipsychotic use, it is unclear whether pregnancy will occur and, if so, how long the time to pregnancy will be. Beyond the perinatal period, relapse risks after tapering of antipsychotics in patients with bipolar disorder have been reported as increased in some studies but not increased in other studies.^6,7,8,9,34^ In the period after delivery, women with bipolar disorder face a high risk of psychiatric relapse. Therefore, prophylactic medication during the postpartum period remains indicated.^30^ We recommend that patients and clinicians consider either lithium, which has the most robust evidence for postpartum prophylaxis, or medications that have demonstrated benefit for the individual.

### Strengths and Limitations

Our study is based on a representative cohort of all pregnant women in Denmark and Sweden, making it among the largest studies on the topic to date. By using data from 2 countries with universal health care access, we minimized selection bias that might affect studies from insurance-based systems. We restricted our analysis to women with diagnosed primary psychotic disorders or bipolar disorder to create homogeneous comparison groups and minimize confounding by indication, focusing on populations for whom antipsychotic maintenance represents core treatment rather than adjunctive intervention. We obtained information on psychiatric diagnoses from registers in the 2 countries. These registers have been extensively validated, with positive predictive values exceeding 85% to 95% for major psychiatric disorders, including bipolar disorder and schizophrenia-spectrum illnesses, and κ statistics ranging from 0.74 to 0.87 for diagnostic agreement.^35,36,37^ Our use of PSM enabled reasonable comparability between discontinuation and continuation groups.

Several limitations should be noted. First, prescription fills do not indicate actual medication use. While we reduced potential misclassification by limiting our analyses to women with at least 2 antipsychotic prescriptions before pregnancy, some degree of misclassification likely remains. We estimated medication coverage using defined daily doses with a grace period. Since actual dose varies between individuals, misclassification of continuation status may have biased estimates toward the null. Second, our PSM approach involved tradeoffs between methodological rigor and sample size. While achieving balanced comparison groups, we necessarily excluded women without suitable matches, which may have limited the generalizability of our findings. Third, we examined antipsychotics as a group, without distinguishing specific agents, switches, or dose changes, although clinical guidelines generally recommend maintaining stable regimens throughout the perinatal period to minimize the risk of destabilization. Fourth, we captured only severe relapses requiring hospitalization because we lacked data on milder symptoms typically managed in outpatient settings, reasons for discontinuation, and tapering strategies.^38^ We were not able to include outpatient visits to define relapse because doing so might introduce misclassification given that such contacts often reflect routine follow-up visits rather than true relapse.

Fifth, residual confounding in either direction remains possible—for example, if women with more unstable illness were less likely to adhere to treatment, or if those with milder illness were more likely to discontinue—although our matching on multiple disease severity proxies should have partially addressed this concern. Sixth, the limited number of matched pairs, particularly for bipolar disorder, constrained precision; thus, we focused on the direction and magnitude of risk estimates rather than strict statistical significance. It is also important to note the substantial heterogeneity across bipolar I disorder, bipolar II disorder, and bipolar spectrum disorders not otherwise specified. Our sample size was insufficient to support more nuanced, subtype-specific analyses, especially for bipolar I disorder. Finally, our study was restricted to pregnancies resulting in live births, which may introduce selection bias. Women who relapsed before pregnancy may have postponed pregnancy or experienced difficulties conceiving and, therefore, would not be captured in our cohort. In addition, pregnancy losses are often incompletely recorded in register data. The association between antipsychotic discontinuation and relapse in these populations after preconception consultation would be more appropriately examined in prospective clinical cohorts that follow women planning for pregnancy.

## Conclusions

In this cohort study, women with primary psychotic disorders were at an increased risk of relapse following antipsychotic discontinuation during pregnancy. Findings suggested potential risk associated with prepregnancy discontinuation as well, although this estimate warrants cautious interpretation. For women with bipolar disorder, discontinuation did not have an elevated risk estimate for severe psychiatric relapse, although this finding should be interpreted cautiously given the limited sample size and inability to capture milder episodes. Women with psychotic disorders or bipolar disorder are at an increased risk of both severe and nonsevere relapse during the postpartum period, and clinical recommendations to use prophylactic medication during the postpartum period remain unchanged.

## Notes

Supplement 1. **eMethods.** Definition of Inpatient or Emergency Room Visit Using the Danish National Registers **eTable 1.** The ICD-8 or ICD-10 Codes for Subgroup Diagnosis of Psychiatric Disorders **eTable 2.** Hazard Ratios of Severe Psychiatric Relapse in the Perinatal Period Associated With Antipsychotic Discontinuation Before Pregnancy **eTable 3.** Hazard Ratios of Severe Psychiatric Relapse in the Perinatal Period Associated With Antipsychotic Discontinuation During Pregnancy **eTable 4.** Hazard Ratios for Severe Psychiatric Relapse During the Perinatal Period Linked to Antipsychotic Discontinuation in Psychotic Disorders in Denmark (Inpatient or Emergency Room Visit, 60-day Grace Period) **eTable 5.** Hazard Ratios of Severe Psychiatric Relapse in the Perinatal Period Associated With Antipsychotic Discontinuation Before Pregnancy (30-day Grace Period) **eTable 6.** Hazard Ratios of Severe Psychiatric Relapse in the Perinatal Period Associated With Antipsychotic Discontinuation During Pregnancy (30-day Grace Period) **eFigure 1.** The Graphical Depiction of the Timeline for Assessing Exposure, Outcomes, and Covariates for Generating the Propensity Score **eFigure 2.** The Distribution of Propensity Scores of Discontinuing Antipsychotics Before Pregnancy versus Continuation Before Matching **eFigure 3.** The Distribution of Propensity Scores of Discontinuing Antipsychotics Before Pregnancy versus Continuation Before Matching **eFigure 4.** Illustration of the Follow-up in Antipsychotic Prepregnancy Discontinuation and Pregnancy Discontinuation, and Their Matched Continuation **eFigure 5.** Standardized Mean Difference in Covariates Before and After Propensity Score Matching in Prepregnancy Discontinuation versus Continuation **eFigure 6.** Standardized Mean Difference in Covariates Before and After Propensity Score Matching in Pregnancy Discontinuation versus Continuation

Supplement 2.
Data Sharing Statement
