# Supplementary material for: Risk of Relapse in Psychotic and Bipolar Disorders After Prenatal Antipsychotic Discontinuation
Source: JAMA Netw Open. 2026 Mar 27;9(3):e260682. doi: 10.1001/jamanetworkopen.2026.0682 (PMC13032156; doi:10.1001/jamanetworkopen.2026.0682)
Supplement: Supplement 1. — eMethods. Definition of Inpatient or Emergency Room Visit Using the Danish National Registers eTable 1. The ICD-8 or ICD-10 Codes for Subgroup Diagnosis of Psychiatric Disorders eTable 2. Hazard Ratios of Severe Psychiatric Relapse in the Perinatal Period Associated With Antipsychotic Discontinuation Before Pregnancy eTable 3. Hazard Ratios of Severe Psychiatric Relapse in the Perinatal Period Associated With Antipsychotic Discontinuation During Pregnancy eTable 4. Hazard Ratios for Severe Psychiatric Relapse During the Perinatal Period Linked to Antipsychotic Discontinuation in Psychotic Disorders in Denmark (Inpatient or Emergency Room Visit, 60-day Grace Period) eTable 5. Hazard Ratios of Severe Psychiatric Relapse in the Perinatal Period Associated With Antipsychotic Discontinuation Before Pregnancy (30-day Grace Period) eTable 6. Hazard Ratios of Severe Psychiatric Relapse in the Perinatal Period Associated With Antipsychotic Discontinuation During Pregnancy (30-day Grace Period) eFigure 1. The Graphical Depiction of the Timeline for Assessing Exposure, Outcomes, and Covariates for Generating the Propensity Score eFigure 2. The Distribution of Propensity Scores of Discontinuing Antipsychotics Before Pregnancy versus Continuation Before Matching eFigure 3. The Distribution of Propensity Scores of Discontinuing Antipsychotics Before Pregnancy versus Continuation Before Matching eFigure 4. Illustration of the Follow-up in Antipsychotic Prepregnancy Discontinuation and Pregnancy Discontinuation, and Their Matched Continuation eFigure 5. Standardized Mean Difference in Covariates Before and After Propensity Score Matching in Prepregnancy Discontinuation versus Continuation eFigure 6. Standardized Mean Difference in Covariates Before and After Propensity Score Matching in Pregnancy Discontinuation versus Continuation [file jamanetwopen-e260682-s001.pdf]

Supplemental Online Content

Liu X, Smout S, Mahjani B, et al. Risk of relapse in psychotic and bipolar disorders after prenatal antipsychotic discontinuation. *JAMA Netw Open*. 2026;9(3):e260682.
doi:10.1001/jamanetworkopen.2026.0682

eMethods. Definition of Inpatient or Emergency Room Visit Using the Danish National Registers
eTable 1. The ICD-8 or ICD-10 Codes for Subgroup Diagnosis of Psychiatric Disorders
eTable 2. Hazard Ratios of Severe Psychiatric Relapse in the Perinatal Period Associated with Antipsychotic Discontinuation Before Pregnancy
eTable 3. Hazard Ratios of Severe Psychiatric Relapse in the Perinatal Period Associated with Antipsychotic Discontinuation During Pregnancy
eTable 4. Hazard Ratios for Severe Psychiatric Relapse During the Perinatal Period Linked to Antipsychotic Discontinuation in Psychotic Disorders in Denmark (Inpatient or Emergency Room Visit, 60-day Grace Period)
eTable 5. Hazard Ratios of Severe Psychiatric Relapse in the Perinatal Period Associated with Antipsychotic Discontinuation Before Pregnancy (30-day Grace Period)
eTable 6. Hazard Ratios of Severe Psychiatric Relapse in the Perinatal Period Associated with Antipsychotic Discontinuation During Pregnancy (30-day Grace Period)
eFigure 1. The Graphical Depiction of the Timeline for Assessing Exposure, Outcomes, and Covariates for Generating the Propensity Score
eFigure 2. The Distribution of Propensity Scores of Discontinuing Antipsychotics Before Pregnancy versus Continuation Before Matching
eFigure 3. The Distribution of Propensity Scores of Discontinuing Antipsychotics Before Pregnancy versus Continuation Before Matching
eFigure 4. Illustration of the Follow-up in Antipsychotic Prepregnancy Discontinuation and Pregnancy Discontinuation, and Their Matched Continuation
eFigure 5. Standardized Mean Difference in Covariates Before and After Propensity Score Matching in Prepregnancy Discontinuation versus Continuation
eFigure 6. Standardized Mean Difference in Covariates Before and After Propensity Score Matching in Pregnancy Discontinuation versus Continuation

This supplemental material has been provided by the authors to give readers additional information about their work.

eMethods. Definition of Inpatient or Emergency Room Visit Using the Danish National Registers

We classified a visit as an inpatient or emergency room visit if it was a physical visit for psychiatric disorders, and the patient-type variable was set to "inpatient" or "emergency room visit" before 2019. From 2019, the third version of the Danish National Patient Registry was implemented, and emergency visits from 2019 to 2022 were defined as physical visits for psychiatric disorders with patient contact <8 hours and priority "ATA1", and inpatient was defined as a patient contact of ≥8 hours or more (Bernstorff M, Hansen L, Perfalk E, et al. Stability of diagnostic coding of psychiatric outpatient visits across the transition from the second to the third version of the Danish National Patient Registry. Acta Psychiatr Scand. 2022;146:272-283.).

© 2026 Liu X et al. *JAMA Network Open*

eTable 1. The ICD-8 or ICD-10 Codes for Subgroup Diagnosis of Psychiatric Disorders

Name of disorders	ICD-8 codes	ICD-10 codes
Any psychiatric disorders	290–309, excluding 290.09, 290.10, 290.11, 290.18, 290.19, 292.x9, 293.x9, 294.x9, 309.x9	F00–F99, excluding F00-F09 and F70–F79
Substance abuse disorder	291.X9, 294.39, 303.X9, 303.20, 303.28, 303.90, and 304.X9	F10–F19
Schizophrenia and related disorders, abbreviated hereafter as psychotic disorders	295.X9, 296.89, 297.X9, 298.29–298.99, 299.04, 299.05, 299.09, and 301.83	F20–F29
Bipolar disorder	296.19, 296.39, and 298.19	F30–31
Depression	296.09, 296.29, 298.09, and 300.49	F32–33
Other mood disorders, except for bipolar and unipolar disorders	296.X9 and 301.19, excluding 296.09, 296.19, 296.29, and 296.39	F34–39
Neurotic, stress-related, and somatoform disorders	300.X9, 305.X9, 305.68, and 307.99 excluding 300.49	F40–F48
Personality disorders	300.19, 301.49, 301.59, 301.69, 301.79, 301.80, 301.81, 301.82, 301.83, and 301.84	F60–F69
Behavioral and emotional disorders with onset usually occurring in childhood and adolescence	306.X9, and 308.0X	F90–F98

© 2026 Liu X et al. *JAMA Network Open*

eTable 2. Hazard Ratios of Severe Psychiatric Relapse in the Perinatal Period Associated with Antipsychotic Discontinuation before Pregnancy

Antipsychotic discontinuation before pregnancy	No of women	No of events	Person-year	Incidence/100 person-years	Unadjusted hazard ratios (95% CI)	Adjusted hazard ratios (95%CI) [a]
**Psychotic disorders**						
**Denmark**						
Discontinuation	223	27	210.7	12.8	1.12 (0.64–1.94)	1.09 (0.61–1.95)
Continuation	223	24	209.6	11.4	1 (ref)	1 (ref)
**Sweden**						
Discontinuation	144	13	135.5	9.6	1.45 (0.62–3.39)	1.44 (0.78–2.66)
Continuation	144	9	135.3	6.6	1 (ref)	1 (ref)
**Combined HR**	NA	NA	NA	NA	1.24 (0.81–1.91)	1.24 (0.82–1.90)
**Bipolar disorders** [b]						
**Sweden**						
Discontinuation	102	6	97.4	6.1	0.52 (0.19–1.41)	0.50 (0.22–1.11)
Continuation	102	11	92.6	11.9	1 (ref)	1 (ref)

[a] The hazard ratios were adjusted by incorporating the matched pair identifier as a separate stratum in the models.
[b] For bipolar disorders, sufficient statistical power was available only in the Swedish cohort.

© 2026 Liu X et al. *JAMA Network Open*

eTable 3. Hazard Ratios of Severe Psychiatric Relapse in the Perinatal Period Associated with Antipsychotic Discontinuation during Pregnancy

**Antipsychotic discontinuation before pregnancy**	**No of women**	**No of events**	**Person-years**	**Incidence/ 100 person-years**	**Unadjusted hazard ratios (95% CI)**	**Adjusted hazard ratios (95%CI)** [a]
**Psychotic disorders**						
**Denmark**						
Discontinuation	207	31	142.2	21.7	1.76 (0.98–3.15)	1.63 (0.87–3.04)
Continuation	207	18	145.9	12.3	1 (ref)	1 (ref)
**Sweden**						
Discontinuation	121	13	81.2	16.0	1.90 (0.76–4.73)	1.57 (0.79–3.12)
Continuation	121	7	82.6	8.5	1 (ref)	1 (ref)
**Combined HR**	NA	NA	NA	NA	1.82 (1.16–2.85)	1.60 (1.01–2.54)
**Bipolar disorders** [b]						
**Sweden**						
Discontinuation	92	5	67.6	7.4	0.99 (0.36–2.70)	1.00 (0.48–2.10)
Continuation	92	6	65.5	9.2	1 (ref)	1 (ref)

[a] The hazard ratios were adjusted by incorporating the matched pair identifier as a separate stratum in the models.
[b] For bipolar disorders, sufficient statistical power was available only in the Swedish cohort.

© 2026 Liu X et al. *JAMA Network Open*

eTable 4. Hazard Ratios for Severe Psychiatric Relapse during the Perinatal Period Linked to Antipsychotic Discontinuation in Psychotic Disorders in Denmark (Inpatient or Emergency Room Visit, 60-day Grace Period)

**Antipsychotic discontinuation**	**No of women**	**No of events**	**Person-years**	**Incidence/100 person-years**	**Unadjusted hazard ratios (95% CI)**	**Adjusted hazard ratios (95%CI)** [a]
**Prepregnancy**						
Discontinuation	223	33	206.3	16.0	0.93 (0.58–1.50)	0.91 (0.55–1.50)
Continuation	223	35	204.3	17.1	1 (ref)	1 (ref)
**Pregnancy**						
Discontinuation	207	34	142.1	23.9	1.29 (0.78–2.16)	1.30 (0.73–2.33)
Continuation	207	26	145.5	17.9	1 (ref)	1 (ref)

© 2026 Liu X et al. *JAMA Network Open*

eTable 5. Hazard Ratios of Severe Psychiatric Relapse in the Perinatal Period Associated with Antipsychotic Discontinuation before Pregnancy (30-day Grace Period)

Antipsychotic discontinuation before pregnancy	No of women	No of events	Person-years	Incidence/100 person-years	Unadjusted hazard ratios (95% CI)	Adjusted hazard ratios (95%CI) [a]
**Psychotic disorders**						
**Denmark**						
Discontinuation	108	15	100.9	14.9	1.72 (0.75–3.93)	1.75 (0.73–4.17)
Continuation	108	9	103.9	8.7	1 (ref)	1 (ref)
**Sweden**						
Discontinuation	73	7	70.2	10.0	1.46 (0.64–3.34)	1.14 (0.56–2.35)
Continuation	73	6	70.6	8.5	1 (ref)	1 (ref)
**Combined HR**	NA	NA	NA	NA	1.58 (0.88–2.84)	1.36 (0.78–2.36)
**Bipolar disorders** [b]						
**Sweden**						
Discontinuation	41	1	40.1	2.5	0.24 (0.03–2.13)	0.25 (0.04–1.52)
Continuation	41	4	37.9	10.6	1 (ref)	1 (ref)

[a] The hazard ratios were adjusted by incorporating the matched pair identifier as a separate stratum in the models.
[b] For bipolar disorders, sufficient statistical power was available only in the Swedish cohort.

© 2026 Liu X et al. *JAMA Network Open*

eTable 6. Hazard Ratios of Severe Psychiatric Relapse in the Perinatal Period Associated with Antipsychotic Discontinuation during Pregnancy (30-day Grace Period)

Antipsychotic discontinuation during pregnancy	No of women	No of events	Person-years	Incidence/100 person-years	Unadjusted hazard ratios (95% CI)	Adjusted hazard ratios (95%CI) [a]
**Psychotic disorders**						
**Denmark**						
Discontinuation	78	9	45.3	19.9	1.56 (0.60–4.02)	1.14 (0.41–3.15)
Continuation	78	9	68.8	13.1	1 (ref)	1 (ref)
**Sweden**						
Discontinuation	72	5	51.7	9.7	0.84 (0.37–1.90)	0.78 (0.38 – 1.57)
Continuation	72	5	50.8	9.8	1 (ref)	1 (ref)
**Combined HR**	NA	NA	NA	NA	1.09 (0.59–2.03)	0.88 (0.49–1.58)
**Bipolar disorders** [b]						
**Sweden**						
Discontinuation	40	4	27.7	14.5	0.99 (0.25–3.89)	1.00 (0.38–2.66)
Continuation	40	4	27.4	14.6	1 (ref)	1 (ref)

[a] The hazard ratios were adjusted by incorporating the matched pair identifier as a separate stratum in the models.
[b] For bipolar disorders, sufficient statistical power was available only in the Swedish cohort.

© 2026 Liu X et al. *JAMA Network Open*

eFigure 1. The Graphical Depiction of the Timeline for Assessing Exposure, Outcomes, and Covariates for Generating the Propensity Score

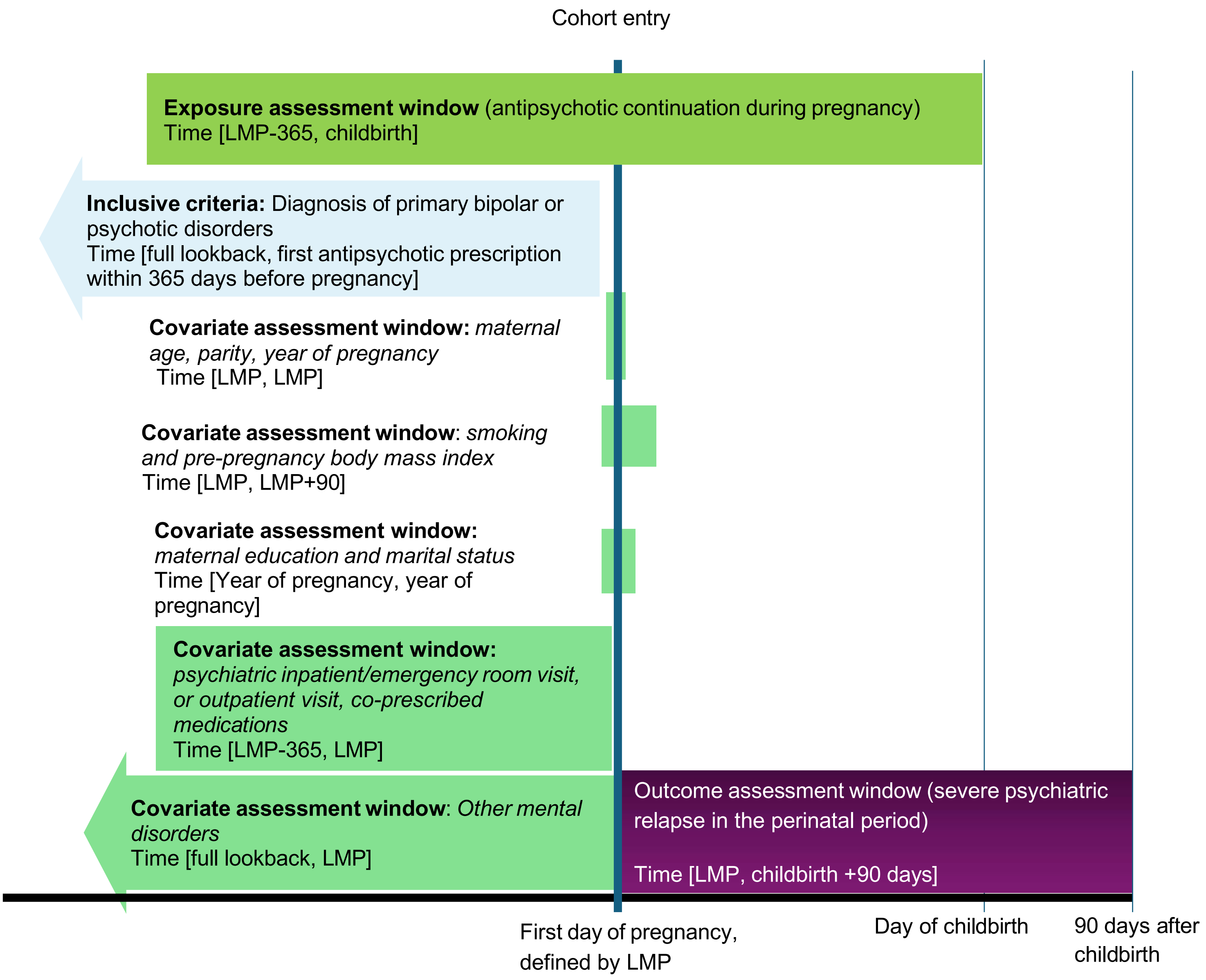

© 2026 Liu X et al. *JAMA Network Open*

eFigure 2. The Distribution of Propensity Scores of Discontinuing Antipsychotics Before Pregnancy versus Continuation Before Matching

eFigure 2a. Psychotic disorders (Denmark)

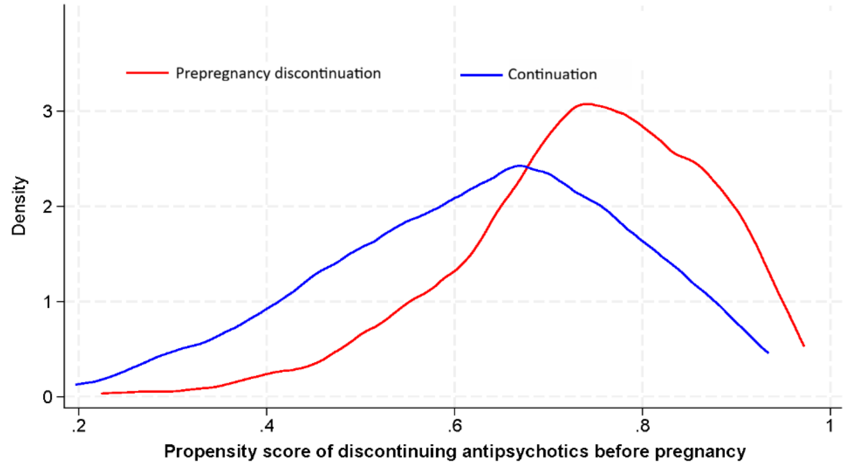

eFigure 2b. Psychotic disorders (Sweden)

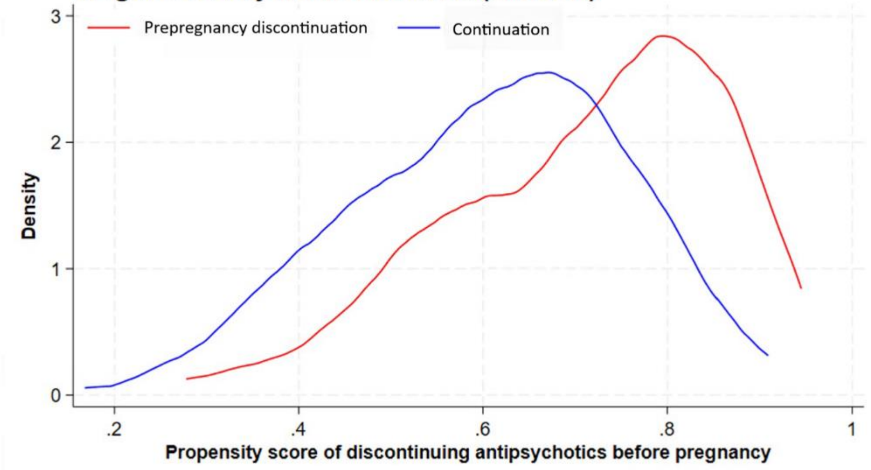

eFigure 2c. Bipolar disorder (Sweden)

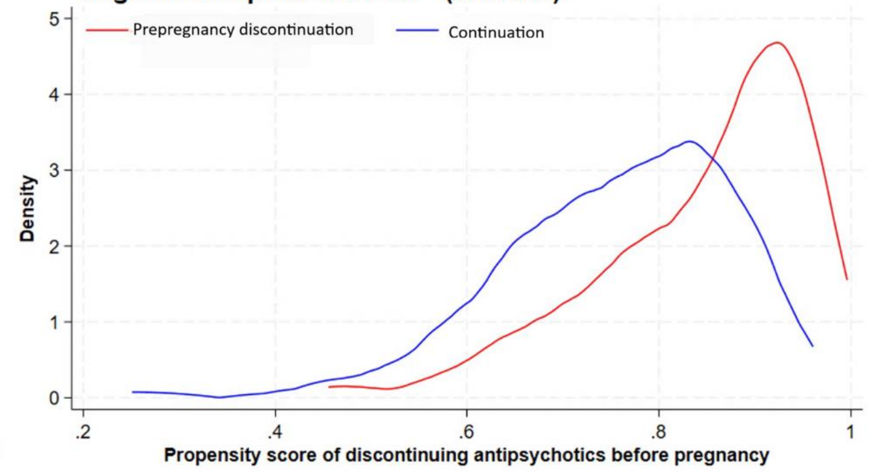

© 2026 Liu X et al. *JAMA Network Open*

eFigure 3. The Distribution of Propensity Scores of Discontinuing Antipsychotics Before Pregnancy versus Continuation Before Matching

eFigure 3a. Psychotic disorders(Denmark)

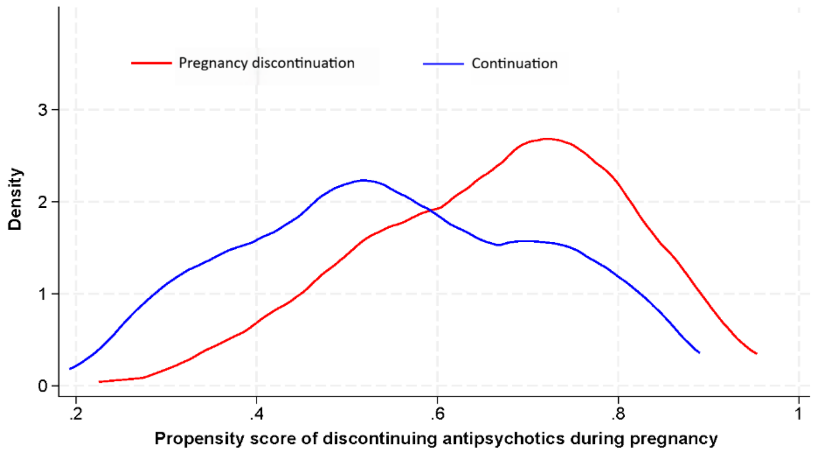

eFigure 3b. Psychotic disorders (Sweden)

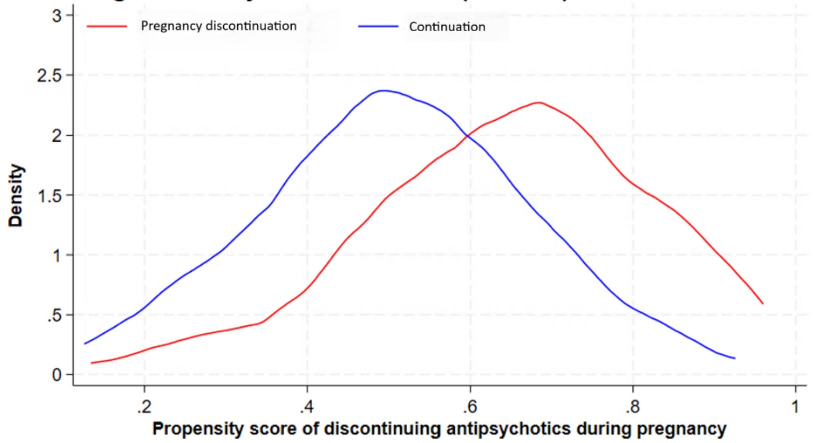

eFigure 3c. Bipolar disorder (Sweden)

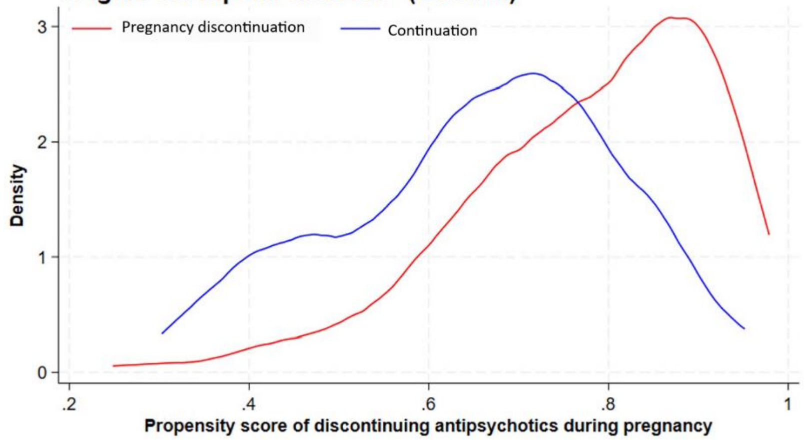

© 2026 Liu X et al. *JAMA Network Open*

eFigure 4. Illustration of the Follow-up in Antipsychotic Prepregnancy Discontinuation and Pregnancy Discontinuation, and their Matched Continuation

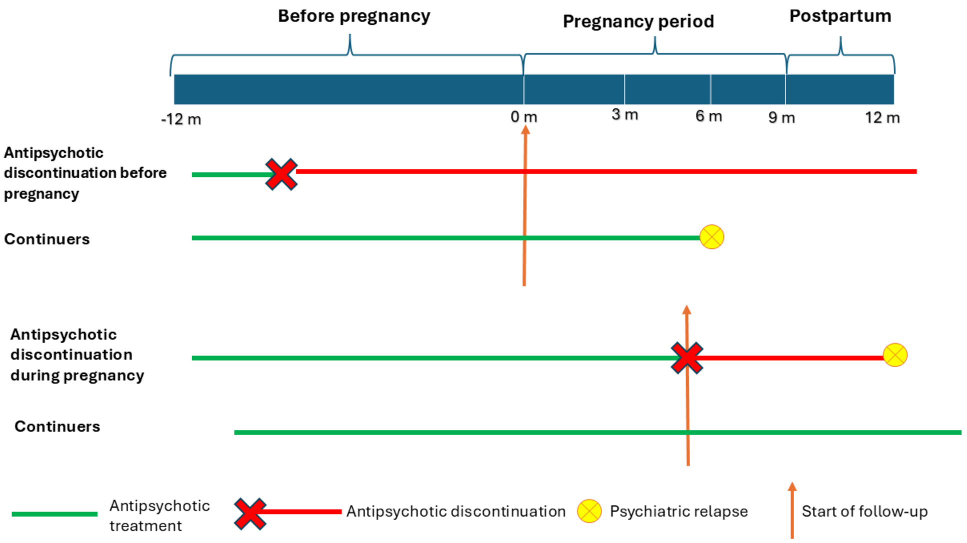

© 2026 Liu X et al. *JAMA Network Open*

eFigure 5. Standardized Mean Difference in Covariates Before and After Propensity Score Matching in Prepregnancy Discontinuation versus Continuation

eFigure 5a. Psychotic disorders (Denmark)

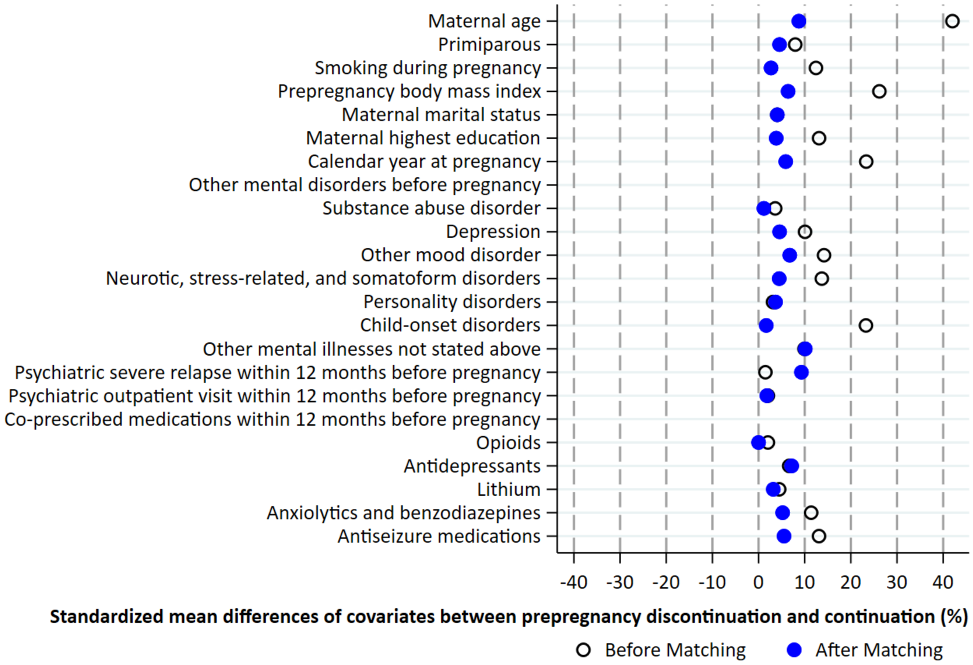

eFigure 5b. Psychotic disorders (Sweden)

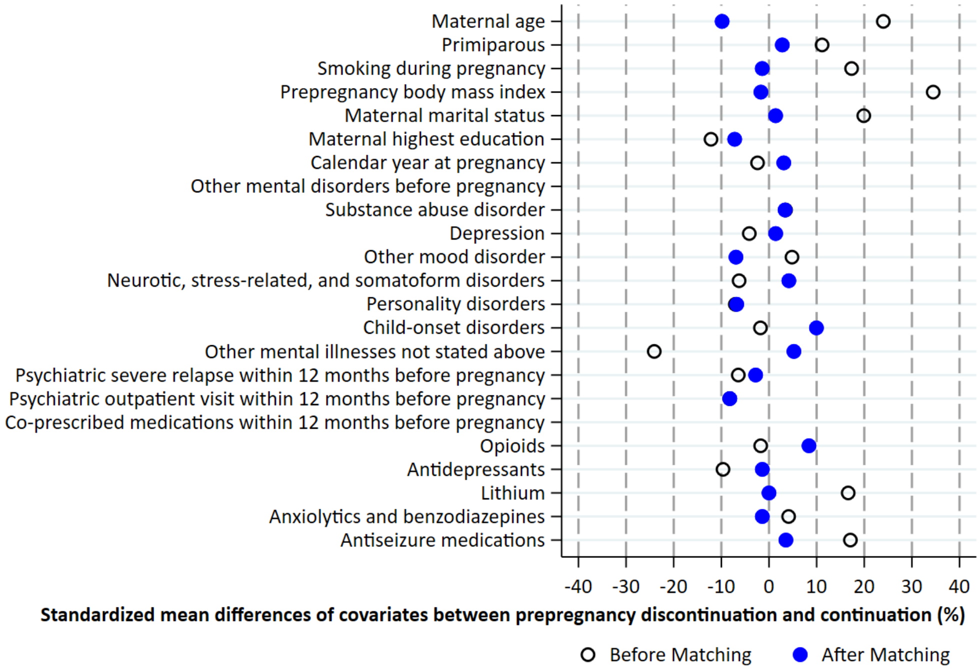

© 2026 Liu X et al. *JAMA Network Open*

eFigure 5c. Bipolar disorders (Sweden)

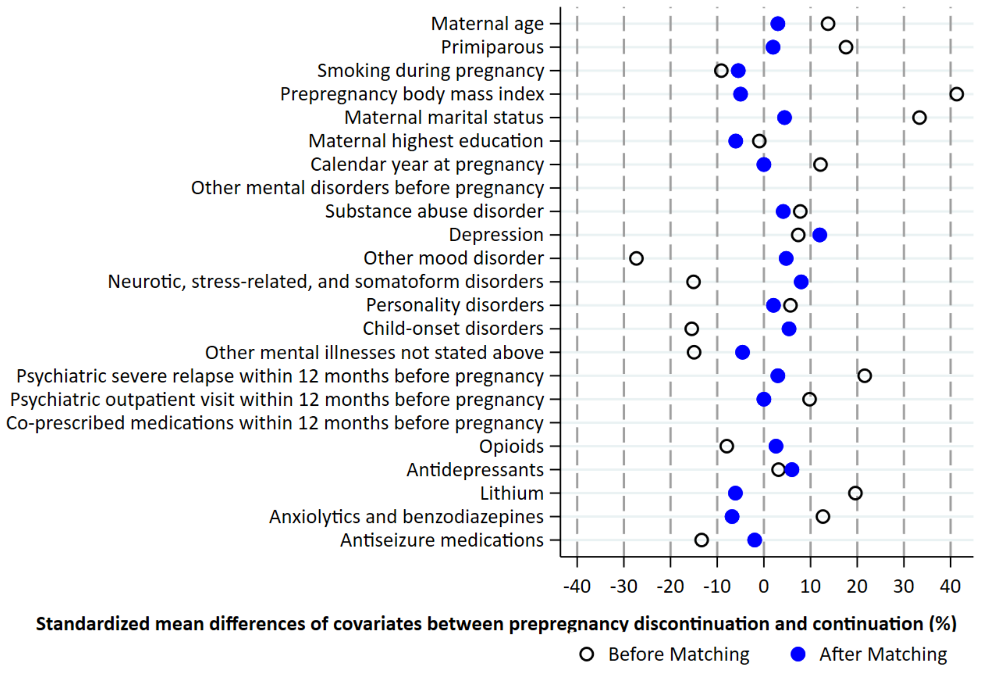

© 2026 Liu X et al. *JAMA Network Open*

eFigure 6. Standardized Mean Difference in Covariates Before and After Propensity Score Matching in Pregnancy Discontinuation versus Continuation

eFigure 6a. Psychotic disorders (Denmark)

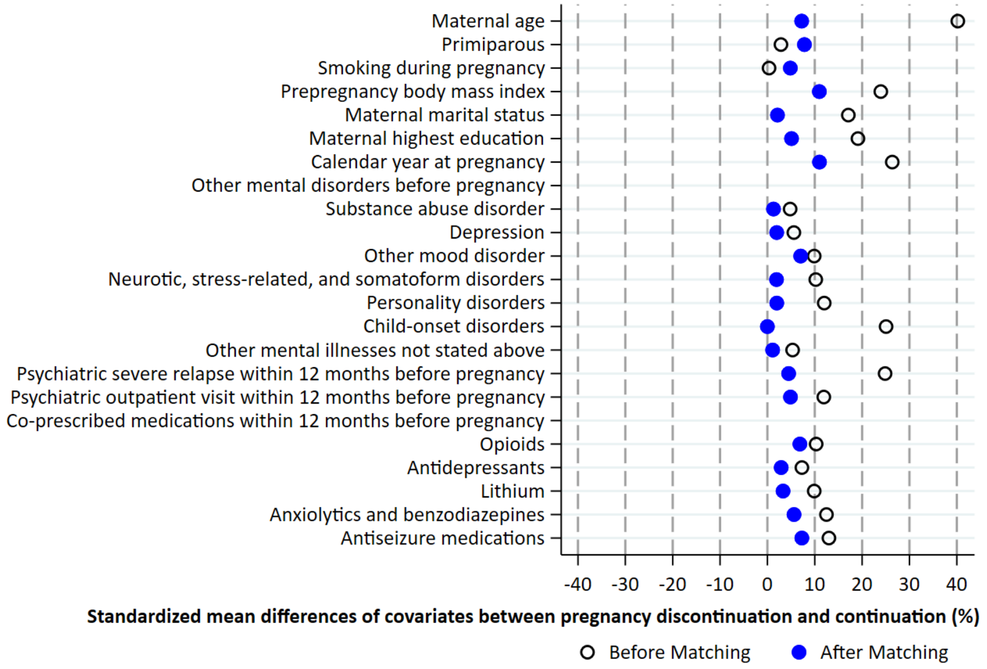

eFigure 6b. Psychotic disorders (Sweden)

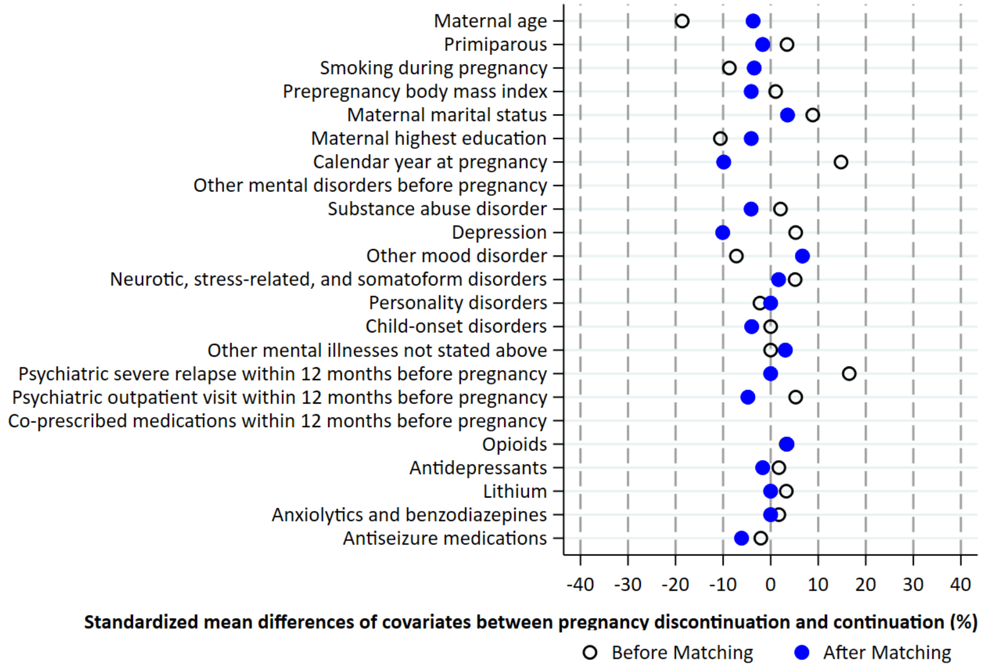

© 2026 Liu X et al. *JAMA Network Open*

eFigure 6c. Bipolar disorder (Sweden)

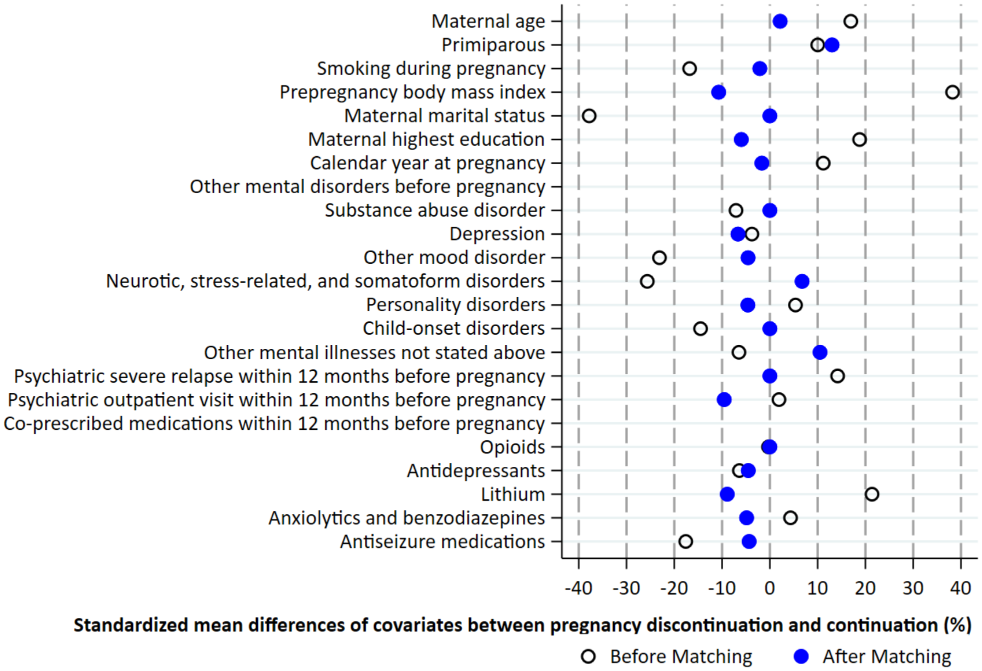

© 2026 Liu X et al. *JAMA Network Open*
