# Supplementary material for: Risk of Relapse in Psychotic and Bipolar Disorders After Prenatal Antipsychotic Discontinuation
Source: JAMA Netw Open. 2026 Mar 27;9(3):e260682. doi: 10.1001/jamanetworkopen.2026.0682 (PMC13032156; doi:10.1001/jamanetworkopen.2026.0682)
Supplement: Supplement 2. — Data Sharing Statement [file jamanetwopen-e260682-s002.pdf]

Data Sharing Statement

Liu. Risk of Relapse in Psychotic and Bipolar Disorders After Prenatal Antipsychotic Discontinuation. *JAMA Netw Open*. Published March 13, 2026. doi:10.1001/jamanetworkopen.2026.0682

Data
Data available: No

Additional Information
Explanation for why data not available: According to Danish data protection legislation, individual-level data cannot be made publicly available; only aggregated data may be presented. All data are stored on a secure platform at Statistics Denmark and are accessible only to authorized personnel. The analytical plan has been registered on the Open Science Framework (OSF) and will be made publicly available upon acceptance of the manuscript.
